# Rapid and Sensitive Quantification of Intracellular Glycyl-Sarcosine for Semi-High-Throughput Screening for Inhibitors of PEPT-1

**DOI:** 10.3390/pharmaceutics13071019

**Published:** 2021-07-03

**Authors:** Teresa von Linde, Gzona Bajraktari-Sylejmani, Walter E. Haefeli, Jürgen Burhenne, Johanna Weiss, Max Sauter

**Affiliations:** Department of Clinical Pharmacology and Pharmacoepidemiology, Heidelberg University Hospital, Im Neuenheimer Feld 410, 69120 Heidelberg, Germany; t.linde@stud.uni-heidelberg.de (T.v.L.); gzona.bajraktari-sylejmani@med.uni-heidelberg.de (G.B.-S.); Walter-Emil.Haefeli@med.uni-heidelberg.de (W.E.H.); juergen.burhenne@med.uni-heidelberg.de (J.B.); johanna.weiss@med.uni-heidelberg.de (J.W.)

**Keywords:** PEPT-1, peptide transport, screening, tandem mass spectrometry, UPLC, glycyl-sarcosine

## Abstract

The peptide transporter PEPT-1 (SLC15A1) plays a major role in nutritional supply with amino acids by mediating the intestinal influx of dipeptides and tripeptides generated during food digestion. Its role in the uptake of small bioactive peptides and various therapeutics makes it an important target for the investigation of the systemic absorption of small peptide-like active compounds and prodrug strategies of poorly absorbed therapeutics. The dipeptide glycyl-sarcosine (Gly-Sar), which comprises an *N*-methylated peptide bond that increases stability against enzymatic degradation, is widely utilized for studying PEPT-1-mediated transport. To support experiments on PEPT-1 inhibitor screening to identify potential substrates, we developed a highly sensitive Gly-Sar quantification assay for Caco-2 cell lysates with a dynamic range of 0.1 to 1000 ng/mL (lower limit of quantification 0.68 nM) in 50 µL of cell lysate. The assay was validated following the applicable recommendations for bioanalytic method validation of the FDA and EMA. Sample preparation and quantification were established in 96-well cell culture plates that were also used for the cellular uptake studies, resulting in a rapid and robust screening assay for PEPT-1 inhibitors. This sample preparation principle, combined with the high sensitivity of the UPLC-MS/MS quantification, is suitable for screening assays for PEPT-1 inhibitors and substrates in high-throughput formats and holds the potential for automation. Applicability was demonstrated by IC_50_ determinations of the known PEPT-1 inhibitor losartan, the known substrates glycyl-proline (Gly-Pro), and valaciclovir, the prodrug of aciclovir, which itself is no substrate of PEPT-1 and consequently showed no inhibition in our assay.

## 1. Introduction

The human peptide transporter PEPT-1 (SLC15A1) is predominantly expressed at the apical surface of enterocytes at the brush border membrane of the small intestine and mediates the uptake of dietary dipeptides and tripeptides [1,2,3]. PEPT-1 is a proton-dependent symporter, which translocates protons together with dipeptides and tripeptides, while the proton gradient is maintained by the sodium/hydrogen exchanger 3 (NHE3) [4,5,6,7]. As a high-capacity, low-affinity transporter, PEPT-1 has a high substrate promiscuity and accepts almost all physiological dipeptides and tripeptides as substrates with affinity constants mostly between 0.1 and 0.5 mM [6,7]. PEPT-1 consists of 700–730 amino acids arranged in 12 transmembrane α-helical domains that form a V-shaped transporter [6]. The transporter alternates in a scissor-like motion between an inward-open and outward-open confirmation to enable access to the central substrate binding site from different sides of the brush border membrane [8,9,10]. Besides its physiological importance in peptide uptake for nutritional supply with amino acids, PEPT-1 also affects the pharmacokinetic properties of small, peptide-like therapeutics by mediating their uptake from the intestinal lumen after oral administration [7]. Accordingly, systemic availability of several β-lactam antibiotics and angiotensin-converting enzyme inhibitors, as well as certain antiviral and antitumor agents, is attributed to PEPT-1 [7]. The low substrate selectivity makes all therapeutics that are structurally and sterically similar to dipeptides or tripeptides potential substrates of PEPT-1. Therefore, PEPT-1 is the subject of investigation into the systemic absorption of peptide-like drugs as well as in studies on associated drug–drug or food–drug interactions. Additionally, prodrug strategies targeting PEPT-1 are an important approach to improve the oral bioavailability of sufficiently small, poorly absorbed therapeutics [11,12,13,14,15,16,17]. To support such medicinal chemistry prodrug developments and identify therapeutics that are potential PEPT-1 substrates likely to suffer from drug–drug or food–drug interactions during intestinal absorption, a rapid screening for PEPT-1 substrates is of high interest, favorably in high-throughput formats.

Because all PEPT-1 substrates are competitive inhibitors of the transporter, a first step in identifying PEPT-1 substrates is the evaluation of their inhibition of PEPT-1-mediated intracellular uptake of known specific substrates. These investigations may be followed by IC_50_ determinations to provide a robust evaluation of PEPT-1 inhibition potency. To ensure that determined IC_50_ values of competitive inhibitors match the corresponding inhibitory constants (*K_i_*), the applied substrate concentration needs to substantially undercut the pertinent *K_m_*, which necessitates highly sensitive quantification [18], especially when low cell numbers are used. To investigate PEPT-1-mediated transport, the determination of changes in the uptake of the dipeptide glycyl-sarcosine (Gly-Sar) has become the gold standard. Gly-Sar is particularly useful because of its high stability against intracellular degradation by ubiquitous di-peptidases, which results from *N*-methylation of the amide bond of the dipeptide. In addition, it has a sufficiently high affinity for PEPT-1 with a *K_m_* of 860 µM [19].

Due to the primary expression of PEPT-1 in the intestinal brush border membrane, we considered the colorectal adenocarcinoma cell line Caco-2 a suitable cellular model to study PEPT-1 uptake. Previous studies have already demonstrated substantial PEPT-1 specific uptake of Gly-Sar in this cell line [12,20,21]. Further, Caco-2 cells form monolayers and subsequently undergo spontaneous enterocytic differentiation into a small intestinal phenotype and, therefore, are a popular model to study drug absorption in the gastrointestinal tract [22]. In addition, it has been demonstrated that Caco-2 cells express considerable levels of human PEPT-1 [23]. Typically, Caco-2 cells are cultured for approximately 21 d to reach a fully differentiated state exhibiting maximal but, at the same time, heterogeneous transporter expression [24,25,26].

Several quantification methods based on LC-MS for intracellular Gly-Sar have been reported previously [20,26,27]. The lowest previously reported lower limit of quantification (LLOQ) was 1 ng/mL in 50 µL cell homogenate relying on cell experiments in formats down to 24-well plates [20]. These previously established methods all involved sample transfer steps of the cells or cell lysates to tubes, as well as sample workup with sonication or solvent evaporation rendering the sample processing relatively laborious. In the present study, we developed an assay for intracellular Gly-Sar quantification with an LLOQ of 0.1 ng/mL in 50 µL of cell homogenate. We aimed at establishing a highly sensitive quantification for concurrently reducing the required number of cells in uptake studies and measure PEPT-1-mediated uptake shortly after monolayer formation (lower transporter expression), which considerably reduces required resources compared to previous methods. For this purpose, we developed a time-efficient sample preparation principle in 96-well formats, which allows for rapid and robust screening for PEPT-1 inhibitors in a semi-high-throughput scale without sample transfer. The feasibility of the assay for identification of PEPT-1 inhibitors was demonstrated by determining IC_50_ values of the known PEPT-1 substrates glycyl-proline (Gly-Pro) and valaciclovir [21], the reported inhibitor losartan [28], and the lack of inhibition by the non-substrate parental compound of valaciclovir, aciclovir.

## 2. Materials and Methods

### 2.1. Drugs, Chemicals, Solvents, and Materials

Caco-2 cells were provided by the American Type Culture Collection (Manassas, VA, USA). Dulbecco’s modified Eagle’s Medium (DMEM), MEM non-essential amino acid solution (NEAS), fetal bovine serum (FBS), penicillin-streptomycin, L-glutamine solution, Dulbecco’s phosphate-buffered saline (PBS), trypsin-EDTA solution, Gly-Sar, Gly-Pro, Boc-Gly-OSu, and 2-(*N*-morpholino)ethanesulfonic acid (MES) were obtained from Sigma-Aldrich (Munich, Germany). Losartan potassium was purchased from Santa Cruz Biotechnology (Dallas, TX, USA) and [^13^C_3_]-sarcosine from Toronto Research Chemicals (North York, ON, Canada). Ammonia solution (25%), *N*,*N*-dimethylformamide (DMF), and trifluoroacetic acid (TFA) were obtained from Merck (Darmstadt, Germany). *tert*-butyl methyl ether (TBME) was provided by VWR International (Darmstadt, Germany). Purified water was produced using an arium® mini (Sartorius, Göttingen, Germany) ultrapure water system. Remaining reagents and solvents, methanol (MeOH), acetonitrile (ACN), and formic acid (FA) were purchased from Biosolve (Valkenswaard, The Netherlands) in the highest purity available. Aprotinin and pepstatin A were purchased from AppliChem (Darmstadt, Germany), leupeptin, aciclovir, and valaciclovir hydrochloride were obtained from Biomol (Hamburg, Germany), whereas pefabloc was received from Carl Roth (Karlsruhe, Germany). RIPA lysis and extraction buffer and the Pierce™ BCA protein assay kit were purchased from Thermo Fisher Scientific (Darmstadt, Germany). All other chemicals were obtained from Sigma-Aldrich (Munich, Germany) if not stated otherwise.

### 2.2. Cell Culture and Inhibition Experiments

Caco-2 cells were cultivated in DMEM supplemented with 10% FBS, glutamine (2 mM), penicillin (100 U/mL), streptomycin (100 µg/mL), and 1% NEAS. They were kept at 37 °C and 5% CO_2_ and passaged once a week. For validation and uptake experiments, cells were seeded in 96-well plates at a density of 6.25 × 10^4^ cells/cm^2^ and allowed to differentiate for at least 7 d to ensure sufficient PEPT-1 expression. The uptake buffer (MES) was prepared as previously described by Sun et al. [29] and contained 50 mM MES, 136.9 mM NaCl, 5.4 mM KCl, 1.26 mM CaCl_2_, 0.81 mM MgSO_4_ × 7 H_2_O, 0.5 mM Na_2_HPO_4_, 0.44 mM KH_2_PO_4_, 0.42 mM NaHCO_3_, and 5.54 mM glucose. The pH value of the uptake buffer was adjusted to pH = 6.0 using either HCl or NaOH solutions. Gly-Sar uptake was tested after 7, 24, and 28 d of Caco-2 cell differentiation. Because no substantial increase in Gly-Sar uptake was observed after longer differentiation times, uptake and competition studies were conducted after 7 d cultivation. For inhibition studies, cells were washed with MES buffer (100 µL/well) once and uptake was initiated by the addition of 0.1–5000 µM losartan, 10–50,000 µM Gly-Pro, 0.1–5000 µM acyclovir, or 1–50,000 µM valaciclovir followed by 20 µM Gly-Sar, all prepared in MES buffer. After an incubation period of 10 min at 37 °C under gentle agitation, transport was terminated by removing the solution and washing the cells with ice-cold MES buffer (100 µL/well) thrice while the plate was kept on ice. Afterwards, cells were lysed in the 96-well cell culture plate with aqueous NH_4_OH (10%, 50 µL/well) and sample preparation was performed as described below.

### 2.3. Protein Quantification

Protein quantification was conducted using the Pierce™ BCA Protein Assay Kit according to the manufacturer’s instructions. The kit is based on the well-known Biuret reaction, in which copper ions are reduced by proteins. In a second step, two molecules of the added bicinchoninic acid (BCA) chelate the reduced copper cation producing a complex with intense purple color, which was measured spectrophotometrically at a wavelength of 555 nm with a SpectraMax ID3 (Molecular Devices, Munich, Germany). The BCA reaction precisely measures the total protein content in lysates extrapolated from a calibration curve obtained by diluting BSA to create standards. The calibration ranged from 0 to 2000 µg protein/mL with a detection limit of 5 µg/mL. In brief, Caco-2 cells were grown confluent for at least 7 d on a 96-well plate. On the day of the measurement, 20 wells in five different columns of the plate were chosen randomly. Cells were washed with warm MES buffer and then lysed with 100 µL cold RIPA buffer with added protease inhibitors. For cell lysis, samples were kept on ice for 30 min and then centrifuged at 4 °C for 15 min at 15,000 g. 50 µL of the supernatant was used for measurement and each sample was measured in duplicate.

### 2.4. Synthesis of the Internal Standard Glycyl-[^13^C_3_]-Sarcosine

To synthesize the internal standard (IS) glycyl-[^13^C_3_]-sarcosine, isotopically labeled [^13^C_3_]-sarcosine, and Boc-glycine *N*-hydroxysuccinimid-ester (Boc-Gly-OSu), the *N*-protected derivative of glycine with an NHS-ester activated carboxylic terminus were used. In brief, [^13^C_3_]-sarcosine and Boc-Gly-OSu were dissolved in a 1/1 (*v/v*) mixture of phosphate buffer (pH = 8.0) and *N*,*N*-dimethylformamide (DMF) and stirred overnight at room temperature. The first lyophilization resulted in a concentrated solution; therefore, the reaction product was extracted with TBME and water followed by another lyophilization step. Afterwards, the Boc-protective group was removed by treatment with a TFA solution containing 5% water (*v/v*) for 2 h. The dipeptide was precipitated by addition of a 20-fold excess of TBME, and subsequently washed with the similar amount of TBME (30 mL). The identity of the newly synthesized glycyl-[^13^C_3_]-sarcosine was confirmed by high-resolution mass spectrometry on a Xevo G2-XS QTof mass spectrometer (Waters, Milford, MA, USA) with a Z-Spray ESI source using the integrated direct infusion system. The measured mass at *m*/z 150.0971 was in good agreement with the theoretical value of m/z 150.0958 (mass error 8.7 ppm). The IS exhibits a mass difference of 3 Da compared to the analyte, Gly-Sar. The obtained glycyl-[^13^C_3_]-sarcosine solution was diluted 100-fold in H_2_O/ACN (95/5, *v/v*) + 0.1% FA to prepare the IS stock solution.

### 2.5. Preparation of Standard and Quality Control Samples

Calibration and quality control (QC) stock solutions were obtained by separately weighing Gly-Sar in 5 mL volumetric flasks and accurately dissolving it in H_2_O/ACN (95/5, *v/v*) + 0.1% formic acid (FA). The stock solutions were then diluted 100-fold with H_2_O/ACN (95/5, *v/v*) + 0.1% FA and used to prepare calibration standard spike solutions at concentrations of 1, 3, 10, 30, 100, 300, 1000, 3000, and 10,000 ng/mL, which corresponds to sample concentrations of 0.1, 0.3, 1, 3, 10, 30, 100, 300, and 1000 ng/mL. QC spike solutions were prepared accordingly and diluted to 1 (LLOQ), 3 (low QC), 600 (mid QC), and 7500 ng/mL (high QC), which corresponds to QC sample concentrations of 0.1, 0.3, 60, and 750 ng/mL. To obtain the IS spike solution, the IS stock solution was diluted 100-fold in H_2_O/ACN (95/5, *v/v*) + 0.1% FA and further diluted 100-fold in ACN/H_2_O (9/1, *v/v*) + 0.1% FA.

### 2.6. Sample Preparation

Sample preparation was performed directly in the 96-well cell culture plates. Cell lysates (50 µL) were spiked with 25 µL IS spike solution and, depending on the sample type, with either 5 µL of the respective calibration or QC solution (calibration and QC samples) or with 5 µL H_2_O/ACN (95/5, *v/v*) + 0.1% FA for volume compensation (study samples). Subsequently, all samples were filled with 180 µL ACN + 0.1% FA for protein precipitation and centrifuged for 10 min at 800 g. Finally, the 96-well plates were transferred into the Sample Manager for direct injection onto the UPLC-MS/MS system.

### 2.7. Instrumental Analysis Parameters

The quantification of Gly-Sar in cell homogenates was performed on a UPLC-MS/MS system consisting of a triple-stage quadrupole mass spectrometer (Waters Xevo TQ-XS with Z-spray ESI source) and an Acquity Classic UPLC® (Waters, Milford, MA, USA). Chromatographic separation was achieved on a Waters Cortecs UPLC® HILIC column (90 Å, 1.6 µm, 2.1 × 50 mm) heated to 60 °C with the injection volume set to 10 µL and a flow rate of 0.4 mL/min. The mobile phase consisted of H_2_O/ACN (95/5, *v/v*) + 0.1% FA (aqueous eluent; A) and ACN + 0.1% FA (ACN eluent; B). Gly-Sar was eluted with an isocratic composition of 16% A/84% B for 3 min. Subsequently, the conditions were changed to 50% A/50% B within 0.5 min and maintained for 0.5 min to flush the column. Afterwards, the initial ratio of 16% A and 84% B was restored during 0.5 min. The mass spectrometer was operated using an electrospray ionization (ESI) source in the positive ion detection mode. Quantification of Gly-Sar and IS was performed by selected reaction monitoring (SRM) using argon collision gas for collision-induced dissociation (CID). Mass spectrometric parameters were optimized for the detection of Gly-Sar and the IS using the auto-optimization feature IntelliStart of the MassLynx V4.2 system software (Waters, Milford, MA, USA). Optimized mass spectrometric conditions are shown in Table 1.

### 2.8. Method Validation

The assay to quantify Gly-Sar in cell homogenates was validated following the EMA and FDA guidelines on bioanalytical method validation [30,31]. For this purpose, the linearity, accuracy, precision, selectivity, stability, recovery, and matrix effect of the assay were determined in three validation batches, which included eight calibration samples in duplicates and 24 QC samples at four concentrations (LLOQ, low, mid, and high QC) in sixfold determination. Accuracy (%) is the degree of agreement between the determined value and known true value and was subsequently determined by dividing the quantified mean concentration by the nominal concentration. Precision (% CV) describes the degree of agreement between individual measurements of the same analyte and was calculated by dividing the standard deviation by the quantified mean of the measurements. Chromatograms of blank cell lysate samples from six different cell passage numbers and of corresponding spiked samples were assessed for interfering signals at the retention time of Gly-Sar and IS to determine the selectivity of the method. Cell culture plates were used for sample preparations and UPLC-MS/MS analyses without any transfer or extraction of samples. Recovery was evaluated for low, mid, and high QC concentration of Gly-Sar by the comparison of peak areas of QC samples spiked after extraction with the respective peak areas of blank plasma samples spiked after extraction. Matrix effects were determined for low, mid, and high QC concentration of Gly-Sar by the comparison of peak areas of blank plasma samples spiked after extraction with the respective peak areas of matrix-free solvent spiked with the identical amount. Recovery was measured by the comparison of peak areas of QC samples (low, mid, and high) to peak areas of blank plasma samples spiked with the respective amount after extraction. Stability of Gly-Sar in cell lysate was evaluated over the course of 24 h by comparing peak areas of stored samples with their initial values. Gly-Sar integrity was further evaluated in the spike solutions.

### 2.9. Calculations and Statistical Methods

Calibration curves were fitted by the Waters TargetLynx V4.2 software (Waters, Milford, MA, USA) using 1/x^2^-weighted linear regressions of the peak area ratios of Gly-Sar and the IS. General calculations were performed using Microsoft Excel 2010 (Mountain View, CA, USA). IC_50_ values (i.e., the inhibitor concentration, which causes a baseline-normalized half-maximal Gly-Sar uptake) were calculated using the GraphPad Prism 9 software (version 9.1.0; GraphPad Software Inc., La Jolla, CA, USA). Intracellular Gly-Sar concentrations at varying inhibitor concentrations were fitted with nonlinear regression to a four-parameter concentration–response curve (4PL) according to the equation:

$$y=bottom+\frac{Top-Bottom}{1+{\left(\frac{\mathrm{IC}50}{x}\right)}^{HillSlope}}$$

where *y* = intracellular Gly-Sar concentration and *x* = inhibitor concentration.

## 3. Results

### 3.1. Mass Spectrometric and Chromatographic Characteristics

**Figure 1:**
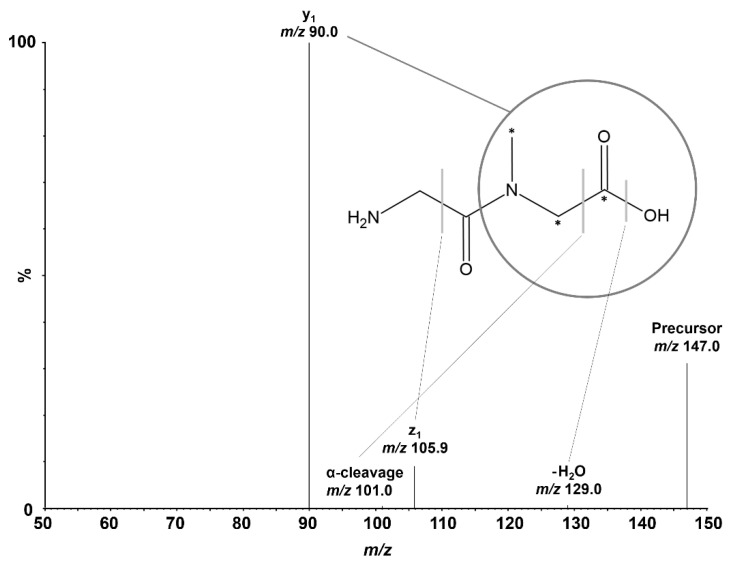
Product ion spectrum of Gly-Sar at a collision energy of 10 V. The structure of Gly-Sar with the positions of the isotopic label in the internal standard marked with asterisks is also depicted. The four investigated dissociation locations are marked by gray bars or a circle, with the circle showing the monitored y_1_-fragment.

Positive electrospray ionization of Gly-Sar (C_5_H_10_N_2_O_3_, 146.14 Da) predominantly formed the [M+H]^+^ precursor ion of Gly-Sar at *m*/z 147.0 and *m*/z 150.0 for the IS. Due to the protonation of the amino terminus of the dipeptide in acidic conditions, Gly-Sar exhibits favorable sensitivity in positive ion mode. During optimization of CID, we investigated the four most intense mass transitions (*m*/z): 147.0 → 90.0 (y_1_), 147.0 → 105.9 (z_1_), 147.0 → 129.0 (non-specific H_2_O-loss), and 147.0 → 101.0 (α-cleavage of the C-terminus that equals non-specific CO and H_2_O loss). Based on its superior intensity and low endogenous background, the selective mass transition of *m*/z 147.0 → 90.0 was chosen for Gly-Sar quantification using SRM. For the IS, the corresponding mass transition of *m*/z 150.0 → 93.0 was monitored. The *N*-methylation of the amide bond of Gly-Sar is crucial for sensitive SRM because the non-methylated analog of the monitored fragment, glycine, similar to most single amino acid product ions, usually shows very high background in cell homogenates. Figure 1 depicts the investigated dissociations of Gly-Sar and the IS. Mass spectrometric conditions were optimized during method development and are listed in Table 1.

**Figure 2:**
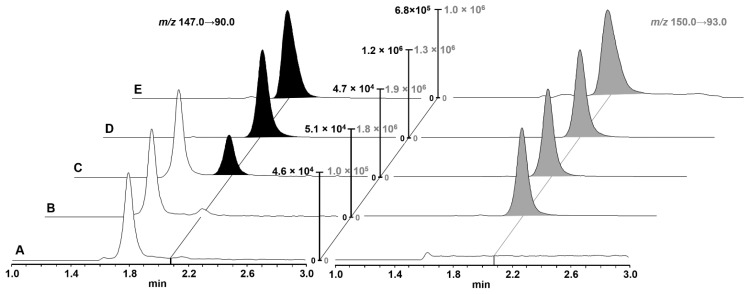
Representative UPLC-MS/MS chromatograms of Gly-Sar in cell lysates. Gly-Sar transition is shown on the left (black filling), and the IS transition is shown on the right (gray filling). Intensities were normalized to the highest signal in the current run: (**A**) blank sample; (**B**) blank lysate with added IS; (**C**) sample at LLOQ concentration; (**D**) sample at mid QC concentration; (**E**) intracellular Gly-Sar concentration after incubation with 20 μM Gly-Sar and 1 mM losartan for 10 min (quantified Gly-Sar concentration: 23.9 ng/mL).

Gly-Sar was chromatographically separated from endogenous substances on a Waters Cortecs HILIC UPLC^®^ column (90 Å, 1.6 µm, 2.1 × 50 mm) using isocratic elution at a composition of 16% aqueous eluent and 84% ACN eluent, which was most efficient in separating endogenous interferences (Figure 2). The method gave reproducible retention times and peak shapes with a width at baseline of 24 s. Representative UPLC chromatograms of cell homogenates with varying Gly-Sar concentrations (blank sample, blank sample spiked with IS, LLOQ, mid QC, and a study sample after 10 min incubation with 20 µM Gly-Sar and 1 mM losartan) are presented in Figure 2.

### 3.2. Sample Preparation

To minimize matrix effects and ensure sensitive and specific quantification of Gly-Sar with reproducible chromatographic characteristics, different sodium-free lysis methods were tested. Cell lysis with 10% NH_4_OH yielded cell homogenates with the lowest viscosity and highest signal intensity compared with lysis using methanol, 1% aqueous triton-X, or 0.1 N hydrochloric acid. Unexpectedly, lysate neutralization proved detrimental to sensitivity, likely due to a resulting high salinity. Sample clean-up was performed with protein precipitation, which was achieved by the addition of 180 µL ACN and subsequent centrifugation. After sample processing, the cell culture plates were directly inserted into the sample manager for Gly-Sar quantification. Because peak areas of samples stored in the autosampler showed <15% deviation from the initial values, we considered Gly-Sar stable during the course of analysis. In addition, Gly-Sar was found stable in spike solutions for at least 9 months, demonstrated by accuracy deviations of <10% of QC solutions prepared from a fresh weighing that were quantified with stored calibration solutions.

### 3.3. Validation Results

The newly developed assay for quantification of intracellular Gly-Sar was validated in compliance with the applicable sections of the FDA and EMA recommendations for bioanalytical method validation [30,31]. No interferences >20% of the LLOQ peak area or >5% of the IS peak area were observed, demonstrating the selectivity of the assay. Linearity was achieved over four orders of magnitude from 0.1 to 1,000 ng/mL, with correlation coefficients (*r*^2^) >0.99 for all calibration curves. Accuracy and precision were consistent with the pertinent EMA and FDA recommendations and are summarized in Table 2.

No carryover was observed after eluent injections following the highest calibration samples. Recovery of the protein precipitation was consistently high, ranging from 75.1 to 83.4% for low to high QC and the IS, while the matrix effects were also consistent over the entire range tested, and were between 29% and 35%; both parameters showed corresponding precisions of ≤10%. The substantial matrix effect is likely induced by the high ammonium and phospholipid content of the processed cell lysate samples. However, the concurrent high sensitivity of the Gly-Sar quantification compensates for the intensity loss from the matrix effect caused by the rapid and well-manageable sample processing.

Normalized to the IS, recoveries and matrix effects were well within required limits, with values from 99.0 and 101.1% and 102.1 and 106.1, respectively.

### 3.4. Application: Inhibition of Gly-Sar Uptake by Gly-Pro, Losartan, Valaciclovir, and Aciclovir

**Figure 3:**
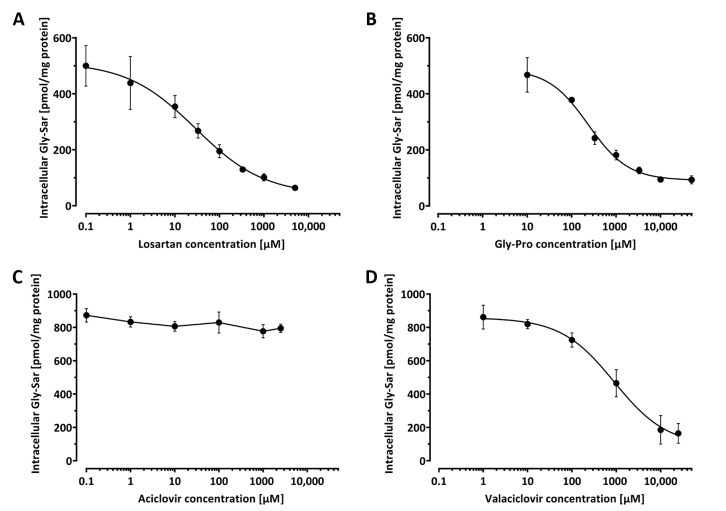
Concentration–response curves on Caco-2 cells for the inhibition of PEPT-1 mediated transport of 20 µM Gly-Sar by losartan (**A**), Gly-Pro (**B**), aciclovir (**C**), and valaciclovir (**D**). Each data point is a mean of three independent experiments, each experiment performed at least in quadruplet determination.

To demonstrate the applicability of our assay for screening of PEPT-1 inhibitors, the effect of Gly-Pro, losartan, valaciclovir, and aciclovir on Gly-Sar uptake was investigated. Protein determination across wells of the 96-well plate verified a highly consistent protein content per well of 64.2 ± 1.8 µg, which corresponds to a variation in protein amount of only ± 2.8%. Caco-2 cells were incubated with 20 µM Gly-Sar in absence and with increasing amounts of the investigated compounds. Because of the substantial transport rate of PEPT-1, intracellular Gly-Sar concentrations were determined after the shortest incubation period that was well manageable, which we regarded to be 10 min. PEPT-1 inhibition properties for Gly-Pro, losartan, valaciclovir, and aciclovir were determined in three independent experiments, each comprising at least quadruplet measurements. Determined IC_50_ values were converted to *K_i_* values using the IC_50_-to-K_i_ tool [18], assuming competitive inhibition and a *K_m_* of 860 µM for Gly-Sar [19]. At pH = 6.0, we observed a concentration-dependent inhibition of Gly-Sar uptake for Gly-Pro, losartan, and valaciclovir. In contrast, aciclovir did not interfere with Gly-Sar uptake (Figure 3). The determined IC_50_ values for Gly-Pro, losartan, and valaciclovir were 257 ± 28 µM, 45.1 ± 15.8 µM, and 894 ± 309 µM, respectively. These correspond to calculated *K_i_* values of 250 µM, 44 µM, and 874 µM, respectively.

## 4. Discussion

Oral drug administration is the preferred route for therapeutics due to its convenience for patients and safe self-administration. However, consistent absorption is an essential prerequisite for oral therapeutics. The simplest way to avoid a high variability in exposure is a high bioavailability. PEPT-1 is a high-capacity transporter highly abundant in the intestine. Therefore, substrates thereof are likely to achieve high intestinal absorption and, as a consequence, high oral bioavailabilities. Small molecule therapeutics may be rendered PEPT-1 substrates by using prodrug approaches relying on chemical modification. Such prodrug developments are especially viable due to the high substrate promiscuity of PEPT-1. Prominent examples for such prodrug developments are the antiviral therapeutics valaciclovir and valganciclovir. These are ester conjugates of the respective parental antiviral drugs and the amino acid valine that exhibit substantially increased oral bioavailabilities due to their absorption via PEPT-1 [12,21,32]. For the purpose of such prodrug developments of poorly absorbed small molecule therapeutics, an efficient identification of PEPT-1 substrates is highly beneficial, especially for screening chemical compound libraries and medicinal chemistry approaches with a high number of test substances. Because all substrates of PEPT-1 are competitive inhibitors of the transporter, determination of their inhibitory properties can be used as a surrogate for substrate screening.

Our developed UPLC-MS/MS assay for intracellular Gly-Sar quantification fulfills the necessary requirements for supporting efficient PEPT-1 inhibitor screenings. In comparison to previously published methods, it is a magnitude more sensitive while concurrently avoiding laborious sample processing and especially sample transfer steps. In contrast to previous methods, we ensured reliable quantification by using an isotopically labeled analog of Gly-Sar as IS that efficiently balanced matrix effects and recovery of the sample processing. This was verified by the very low deviation of the IS-normalized matrix effects and recovery. Our established sample processing directly performed in the cell culture plates renders the assay very rapid and well manageable in high-throughput formats and may even be suitable for automation. However, we consider this methodology as semi-high throughput because the sequential mass spectrometric analysis limits the overall analysis rate. Further, due to the high sensitivity of the quantification assay, the required number of cells in uptake studies can be lowered substantially, which considerably reduces the required resources in regard to cell culture and the especially important required amount of test substance of potential inhibitors. Further, the used Caco-2 cells form monolayers, a characteristic that results in an equal cell number across the wells of the 96-well plate, facilitating accurately comparable results. We could demonstrate this by the measurement of a highly consistent protein content per well. The deviation between wells was very low (<3%) and can be considered neglectable, especially considering the anticipated systematic error of the detection method. As a consequence, if normalization to protein amount is necessary for comparison to other studies, determination of the protein content of few wells can serve for that purpose. In screening experiments, this consistent cell content allows for accurate comparisons of inhibitory power between tested compounds.

We demonstrated the feasibility of our assay for PEPT-1 inhibitor screens by determining the IC_50_ of the four compounds Gly-Pro (dipeptide substrate), losartan (high-affinity inhibitor), valaciclovir (prodrug substrate), and aciclovir (non-substrate) with high replicate numbers, which was facilitated by the used 96-well format. The calculated *K_i_* values of 44 µM, 250 µM, and 874 µM agreed reasonably well with reported *K_i_* values of 24 µM [28], 300 µM [33], and 740 µM [21], for losartan, Gly-Pro, and valaciclovir, respectively. Moreover, as expected, the non-substrate aciclovir did no inhibit the Gly-Sar uptake in our assay. This clearly demonstrates that our newly developed assay is suitable for rapid and well-manageable screening for PEPT-1 inhibitors and further applicable to determinations of pertinent IC_50_ values in high-throughput formats.

Throughout all measured IC_50_ determinations, the ratios of bottom to top of the fitted sigmoidal curves were similar, with a mean of 14.0 ± 6.7%. This non-inhibited (non-PEPT-1 specific) Gly-Sar uptake may source from passive uptake via diffusion or macropinocytosis or may result from amino acid transporters for which Gly-Sar is a substrate such as hPAT1 (SLC36A1) [34]. However, the majority of Gly-Sar uptake (around 86%) is PEPT-1 mediated, which is consistent with previous reports [21]. Nevertheless, short incubation times may be essential in avoiding extensive non-PEPT-1 specific uptake of Gly-Sar. Previous studies showed that Gly-Sar uptake approaches saturation shortly beyond 10 min [26,35]. However, incubation times shorter than 10 min increase assay intrinsic errors and are not well manageable. Thus, we chose 10 min in our assay and due to the high fraction of Gly-Sar uptake that was specific for PEPT-1 in our setting, we regard this incubation time feasible for inhibitor screening.

## 5. Conclusions

We established a highly sensitive intracellular Gly-Sar quantification assay with robust and reliable quantification due to a chemically synthesized isotopically labeled analog as IS. Because of the assay´s high sensitivity, simple and rapid sample processing was established directly in 96-well plates used for cell culture, evading laborious sample workup and transfer steps. This sample preparation procedure is, therefore, suitable to assess PEPT-1 inhibitor properties in high-throughput formats and the assay can serve as a screening tool for potential PEPT-1 substrates.

## Figures and Tables

**Table 1:** Optimized parameters for the MS/MS detection of glycyl-sarcosine and glycyl-[^13^C_3_]-sarcosine in positive heated ESI and SRM.

Parameter	Gly-Sar (glycyl-[^13^C_3_]-sarcosine)
Spray voltage Cone voltage Cone gas flow Source temperature Desolvation gas flow (N_2_) Desolvation temperature MRM transition (*m/*z) Dwell time Collision energy Collision gas flow (Ar)	0.5 kV 30 V 150 L/Hr 150 °C 1000 L/h 500 °C 147.0 → 90.0 (150.0 → 93.0) 100 ms 10 V 0.15 mL/min

ESI, electrospray ionization; SRM, selected reaction monitoring.

**Table 2:** Quality control results of the validation.

		LLOQ	Low QC	Mid QC	High QC
		0.100 ng/mL	0.300 ng/mL	60.0 ng/mL	750 ng/mL
**Within-Batch**					
**1**	Mean [ng/mL]	0.116	0.311	54.3	703
	Accuracy [%]	116.0	103.7	90.6	93.8
	Precision [%CV]	0.704	3.52	3.84	2.76
**2**	Mean [ng/mL]	0.106	0.312	59.2	715
	Accuracy [%]	106.0	104.0	98.7	95.4
	Precision [%CV]	6.05	4.13	2.87	0.854
**3**	Mean [ng/mL]	0.107	0.312	56.7	691
	Accuracy [%]	107.0	104.0	94.4	92.2
	Precision [%CV]	10.3	9.96	3.71	1.81
**Batch-to-Batch**					
	Mean [ng/mL]	0.110	0.311	56.8	702
	Accuracy [%]	110.0	103.7	94.6	93.6
	Precision [%CV]	7.47	5.77	4.93	2.36

CV, coefficient of variation; LLOQ, lower limit of quantification; QC, quality control; *N* = 4 replicates at LLOQ and each QC concentration.

## Data Availability

The data are available from the corresponding author upon reasonable request.
